# The effect of storage time and temperature on the proteomic analysis of FFPE tissue sections

**DOI:** 10.1186/s12014-025-09529-5

**Published:** 2025-02-05

**Authors:** Jennifer M. S. Koh, Erin K. Sykes, Jyoti Rukhaya, Asim Anees, Qing Zhong, Christopher Jackson, Benedict J. Panizza, Roger R. Reddel, Rosemary L. Balleine, Peter G. Hains, Phillip J. Robinson

**Affiliations:** 1 Faculty of Medicine and Health, ProCan®, Children’s Medical Research Institute, The University of Sydney, Westmead, NSW Australia; 2 Department of Otolaryngology, Head and Neck Surgery, Princess Alexandra Hospital, Brisbane, QLD Australia; 3 Queensland Head and Neck Cancer Centre, Princess Alexandra Hospital, Brisbane, QLD Australia; 4 Faculty of Medicine, University of Queensland, Brisbane, QLD Australia; 5 Faculty of Medicine and Health, The Westmead Institute for Medical Research, The University of Sydney, Westmead, NSW Australia

## Abstract

Formalin-fixed paraffin-embedded (FFPE) tissues present an important resource for cancer proteomics. They are more readily available than fresh frozen (FF) tissues and can be stored at ambient temperature for decades. FFPE blocks are largely stable for long-term preservation of tumour histology, but the antigenicity of some proteins in FFPE sections degrades over time resulting in deteriorating performance of immunohistochemistry (IHC). It is not known whether FFPE sections that have previously been cut from blocks and used for liquid chromatography-mass spectrometry (LC–MS) analysis at a later time are affected by storage time or temperature. We determined the stability of FFPE sections stored at room temperature (RT) versus − 80 °C over 48 weeks. The stored sections were processed at different timepoints (n = 11) and compared to sections that were freshly cut from FFPE blocks at each timepoint (controls). A total of 297 sections (rat brain, kidney and liver stored at RT, − 80 °C or freshly cut) were tryptically digested and analysed on TripleTOF 6600 mass spectrometers in data-dependent acquisition (DDA) mode. Kidney and liver digests were also analysed in data-independent acquisition (DIA) mode. The number of proteins and peptides identified by DDA with ProteinPilot and some common post-translational modifications (PTMs) were unaffected by the storage time or temperature. Nine of the most common FFPE-associated modifications were quantified using DIA data and all were unaffected by storage time or temperature. Therefore, FFPE tissue sections are suitable for proteomic studies for at least 48 weeks from the time of sectioning.

**Supplementary Information:**

The online version contains supplementary material available at 10.1186/s12014-025-09529-5.

## Introduction

The application of liquid chromatography-mass spectrometry (LC–MS) methods for proteomic analysis of whole tissues has the potential to advance our understanding of complex diseases such as cancer and to discover novel biomarkers. Tissue specimens from patients are most commonly preserved using the formalin-fixed paraffin-embedded (FFPE) method as this protects samples from auto-proteolysis and stabilises proteins for decades at ambient temperature [1, 2]. FFPE specimens are primarily used for histopathology but also used for immunohistochemistry (IHC) to detect specific proteins that are recognised by antibodies [3, 4]. Clinical diagnostic and treatment decisions can then be made based on the histopathology and immunostaining results.

The use of FFPE samples in molecular biological applications was previously limited by the modifications of macromolecules that occur during formalin fixation [5]. However, the problem of formaldehyde-induced cross-linking has largely been overcome and many studies have extracted nucleic acids [6–11] and proteins [2, 12–18] from FFPE samples that are suitable to be used for downstream analyses. Using FFPE samples for genomic, transcriptomic and proteomic analyses has many advantages such as access to large specimen archives with all types of tissues; many of these archives have associated clinical data including pathology reports, diagnosis, treatment and clinical outcomes [12, 19]. FFPE samples pose fewer biological risks to the operators and are less expensive to store and transport than fresh frozen (FF) samples. The use of FFPE samples facilitates translation of research findings into clinical practice as FFPE is the standard preservation method used worldwide for clinical diagnostic and archival purposes. The quality and quantity of nucleic acids and proteins extracted from FFPE sections cut from tissue blocks that had been stored for at least 10 years were shown not to be compromised by the storage time [1, 3, 20, 21]. Multiple studies have also demonstrated that long-term storage of FFPE tissue blocks did not have significant effects on MS results, and the primary difference between FF and FFPE samples was that the proportion of *C*-terminal lysine to *C*-terminal arginine peptides was lower in FFPE samples [2, 20, 22–24]. Detailed and discriminative information can be derived from both sample types and has the potential for biomarker discovery and validation [15, 19].

What is not known, however, is whether FFPE samples are suitable for mass spectrometric analysis if they are stored for prolonged period as cut sections. Storage of FFPE sections is known to result in degradation of the ability of IHC to detect some, but not all proteins [25–29]. There have also been suggestions that antigenicity can be maintained after cutting of sections by storing them at low temperature and dry conditions so that the results are comparable with that of fresh cut sections [29]. In pathology laboratories, therefore, IHC is preferentially performed on sections that are freshly cut from the FFPE tissue blocks, but for LC–MS proteomic studies, this is rarely practicable. Many pathology departments are unable to release tissue blocks from their custody and instead send sections to the proteomic laboratory, and it may take many months to assemble a sample cohort from multiple sources and then analyse them in a constrained timeframe to mitigate potential bias from technical variations in sample preparation and instrument configuration [28]. Thus, the requirement that all tissue samples for a cohort should be assembled prior to being analysed together means that the period between sectioning from the block and processing to peptides may vary substantially across a sample set. The potential for this difference to impact comparative analyses is unknown.

The aims of this study were to determine the effect of storage time and storage temperature on FFPE tissue sections for LC–MS based proteomic analysis. Our approach was to compare fresh cut sections with sections from the same blocks that had been cut and stored at room temperature (RT) or -80°C for a period of up to 48 weeks. All samples were analysed by data-dependent acquisition (DDA) LC–MS, and a proportion were also analysed in data-independent acquisition (DIA) mode.

## Methods

### Preparation of FFPE tissue sections

#### FFPE tissue sectioning and review

Brain, kidney and liver were harvested from an adult male Wistar rat. Tissues were fixed in 10% (v/v) neutral buffered formalin for 24 h and then transferred to 70% (v/v) ethanol prior to being embedded into paraffin blocks. Use of experimental animal tissues was approved by the Animal Care and Ethics Committee for Children’s Medical Research Institute/Sydney Children’s Hospital Network, Sydney, Australia, approval number C116.

To ensure that there would be sufficient comparable tissues for the entire experiment, three blocks were prepared from each tissue (total n = 9). A section from each tissue block was prepared for each timepoint and storage temperature so that each tissue was represented in triplicate. Tissue blocks were sectioned using a rotary microtome (Leica Biosystems, Nussloch, Germany) to collect a 4 µm section onto a glass slide for haematoxylin and eosin (H&E) staining, followed by 10 µm sections for proteomic analysis. Each 10 µm section was placed into a Barocycler MicroTube (Pressure Biosciences Inc., MA, USA) and then stored in an enclosed tube. Sections were numbered sequentially from the surface of the block. H&E stained slides were scanned to digital images (Zeiss Axio Scan Z1, Oberkochen, Germany) and viewed with Zen 3.1 (Zen lite) viewing software (Zeiss). Tissue size was measured using a manual outline trace and automated area measurement (in mm^2^).

#### Experiment schedule

On day 0, 22 sections were cut from each of the nine tissue blocks for proteomic analysis (total n = 198). Odd numbered samples from each block were stored at RT (around 22°C) and even numbered samples were stored at − 80°C. An H&E slide was prepared from each block surface after every eight 10 µm sections to track changes in tissue area. Tissue blocks were stored at RT throughout the experiment. Eleven experiment days were scheduled on 1, 2, 3, 4, 8, 12, 16, 20, 24, 37 and 48 weeks after day 0. On each experiment day, an H&E slide and a 10 µm section were prepared from each of the nine tissue blocks. These samples were combined with two sets of nine tubes that were stored at RT and − 80°C from day 0 to form a sample set (n = 27).

#### Oropharyngeal tissue sections

Human oropharyngeal tissue sections (10 µm) were prepared at the Princess Alexandra Hospital (PAH, Brisbane, QLD, Australia) with ethics approval obtained through the Metro South Human Research Ethics Committee at PAH (HREC/14/QPAH/54).

### LC–MS analysis of tissue sections

#### Sample preparation

All samples were processed for LC–MS using the Heat ‘n Beat (HnB) method [18]. Paraffin wax was removed from tissue samples with heptane-methanol for 10 min at 30°C then samples were dried for 10 min and suspended in 5% (w/v) sodium deoxycholate (SDC) in 100 mM triethylammonium bicarbonate (TEAB), 4.2 mM tris(2-carboxyethyl)phosphine (TCEP) and 16.75 mM iodoacetamide (IOA) for 7 min at 95°C. Zirconium beads (Benchmark Scientific, NJ, USA), 50 µL of Rapid Digest Buffer (Promega, Alexandria, NSW, Australia), 1 µg of Rapid Trypsin/Lys-C (Promega) and 1 unit of Benzonase nuclease (Sigma-Aldrich, Castle Hill, NSW, Australia) were added prior to 1 min homogenisation in a Beadbug homogeniser (Benchmark Scientific, NJ, USA) at 3,800 rpm. Further lysis and digestion were carried out in a Barocycler 2320EXT (Pressure BioSciences Inc.) for 30 min at 56°C. The digests were acidified with 5 µL of formic acid (FA) to precipitate the SDC before being centrifuged for 15 min (18,000 × *g*, 4°C). Peptides were extracted from the supernatant using Oasis PRiME HLB 1 cc (30 mg sorbent) SPE cartridges (Waters, Rydalmere, NSW, Australia) and their concentration was determined using A280 nm with an Implen nanophotometer NP80 (Implen, München, Germany).

#### DDA MS acquisition

All samples (n = 297) were analysed in DDA mode on multiple mass spectrometers over 48 weeks. Samples from the same experiment day were analysed on the same mass spectrometer in randomised order. An Eksigent nanoLC 425 HPLC (SCIEX, MA, USA) operating in microflow mode, coupled online to a TripleTOF 6600 (SCIEX) was used for all analyses. Peptide digests (2 µg) were spiked with retention time standards (non-human internal spike peptides, Biognosys iRT peptides and PROCAL peptides) and injected onto a C18 trap column (SGE TRAPCOL C18 G203 300 µm × 100 mm) and desalted for 5 min at 8 µL/min with solvent A (0.1% [v/v] FA). The trap column was switched in-line with a reversed-phase capillary column (SGE C18 G203 300 µm × 250 mm, ID 3 µm, 200 Å), maintained at 40 °C. The flow rate was 5 µL/min. The gradient started at 2% solvent B (99.9% [v/v] acetonitrile, 0.1% [v/v] FA) and increased to 10% over 5 min. This was followed by an increase of solvent B to 25% over 60 min, then a further increase to 40% for 5 min. The column was washed with a 4 min linear gradient to 95% solvent B held for 5 min, followed by a 9 min column equilibration step with 98% solvent A. The LC eluent was analysed using the TripleTOF 6600 system equipped with a DuoSpray source and 50 µm internal diameter electrode and controlled by Analyst 1.7.1 software. The following parameters were used: 5500 V ion spray voltage; 25 nitrogen curtain gas; 100 °C TEM; 20 source gas 1; 20 source gas 2. The 90 min DDA acquisition, consisted of a survey scan of 200 ms (TOF–MS) in the range 350–1250 m/z to collect the MS1 spectra and the top 40 precursor ions with charge states from + 2 to + 5 were selected for subsequent fragmentation with an accumulation time of 50 ms per MS/MS experiment for a total cycle time of 2.3 s and MS/MS spectra were acquired in the range 100–2000 m/z.

#### DIA MS acquisition

Kidney (n = 66) and liver (n = 99) samples were analysed in DIA mode after all the samples were processed and analysed by DDA. For the DIA acquisition, peptide spectra were acquired with the LC–MS/MS method described above for DDA, using 100 variable windows. The parameters used were: lower m/z limit 350; upper m/z limit 1250; window overlap (Da) 1.0; CES was set at 5 for the smaller windows, then 8 for larger windows, and 10 for the largest windows. MS2 spectra were collected in the range 100–2000 m/z for 30 ms in high resolution mode and the resulting total cycle time was 3.2 s.

### Data processing and analysis

#### ProteinPilot

DDA data were searched using ProteinPilot version 5.0 (SCIEX) with Paragon against a rat SWISSPROT database (downloaded on 17 July 2019) that included canonical sequences only. It contained 8,071 proteins and 16,142 peptides with manually inserted retention time standards. The following search parameters were used: sample type—identification; Cys alkylation—iodoacetamide; digestion—trypsin; instrument—TripleTOF 6600; search effort—thorough ID; detected protein threshold—0.05 (10.0%), the false discovery rate (FDR) analysis was selected and set at 1%.

#### MSFragger

Raw MS files were converted to mzML format using MSConvertGUI version 3.0 [30] with peak peaking vendor MS = level 1–2. MSFragger version 3.1.1 [31] was run on FragPipe version 14.0, with Philisopher version 3.3.12 and Python version 3.8.3. All DDA data were subjected to an open search using default open search settings with RAM set as 64, parallelism 60 and regular MS was selected. The rat SWISSPROT canonical database used for ProteinPilot search was also used for MSFragger search with decoys added. Fragment mass tolerance and precursor true tolerance were set as 50 ppm. Crystal-C, PeptideProphet, ProteinProphet and PTM-Shepherd were selected and run using defaults for open search. DDA runs from weeks 1, 12, 24, 37 and 48 were subjected to a closed search against the same rat database using default LFQ settings. For MSFragger analysis, the closed search default config was selected, fragment mass tolerance and precursor true tolerance were set as 20 ppm. A list of variable modifications that was identified from the open search which are common for FFPE samples was included in the search and a maximum of three variable modifications was allowed for each peptide (Table 1). PeptideProphet, ProteinProphet and MS1 Quantification were selected and run using defaults for closed search. A spectral library was generated from the search results using EasyPQP.

**Table 1:** Modifications commonly associated with FFPE samples and reported in the literature were identified using an MSFragger open search of DDA runs

Site	Mass Delta	Modification	Max Occurrence
Proline	− 30.0105	Proline oxidation to pyrrolidinone	2
*N*-terminal	12	Formaldehyde adduct	1
Arginine	12	Formaldehyde adduct	2
Lysine	12	Formaldehyde adduct	2
*N*-terminal	14.0156	Methylation	1
Lysine	14.0156	Methylation	2
Methionine	15.9949	Oxidation	2
Proline	15.9949	Oxidation	2
*N*-terminal	26.0156	Acetaldehyde adduct	1
*N*-terminal	27.9949	Formylation	1
Lysine	27.9949	Formylation	2
*N*-terminal	30.0105	Formaldehyde-induced modifications	1
Lysine	30.0105	Formaldehyde-induced modifications	2
Methionine	31.9898	Dihydroxylation	2
Proline	31.9898	Dihydroxylation	2
*N*-terminal	42.0106	Acetylation	1

The peptide terminus or the amino acid residues that are affected, the changes in peptide mass and the maximum number of occurrences within a peptide were specified and included as variable modifications in the closed search of selected DDA runs

#### **DIA-NN**

DIA data were searched using Data-Independent Acquisition by Neural Networks (DIA-NN) version 1.7.12 [32] against the spectral library generated from the closed search on MSFragger. The following settings were used: protease—Trypsin/P with 1 missed cleavage; maximum number of variable modifications—0; *N*-term M excision and C carbamidomethylation were selected; peptide length range 7–30; precursor m/z range 400–1250; fragment ion m/z range 100–2000; precursor FDR set at 1%; cross-run normalisation—RT-dependent; quantification strategy—robust LC (high precision). Fixed modifications were allowed for the search, including the nine FFPE-specific modifications identified from MSFragger open search (Table 1). LOESS normalisation was performed on the peptide matrix before further analysis.

### Statistical analysis

For the statistical analysis of DDA data, paired *t*-test followed by Bonferonni-Dunn’s multiple comparisons was performed using GraphPad Prism version 9.4.0 for Windows (CA, USA). Paired *t*-test was also performed on DIA data using Python modules scipy.stats [33] and statsmodels.stats.multitest [34], with Bonferonni method being selected for *p*-value correction.

### Data availability

The mass spectrometry proteomics data have been deposited to the ProteomeXchange Consortium via the PRIDE [35] partner repository with the dataset identifier PXD054596.

## Results and discussion

**Fig. 1:**
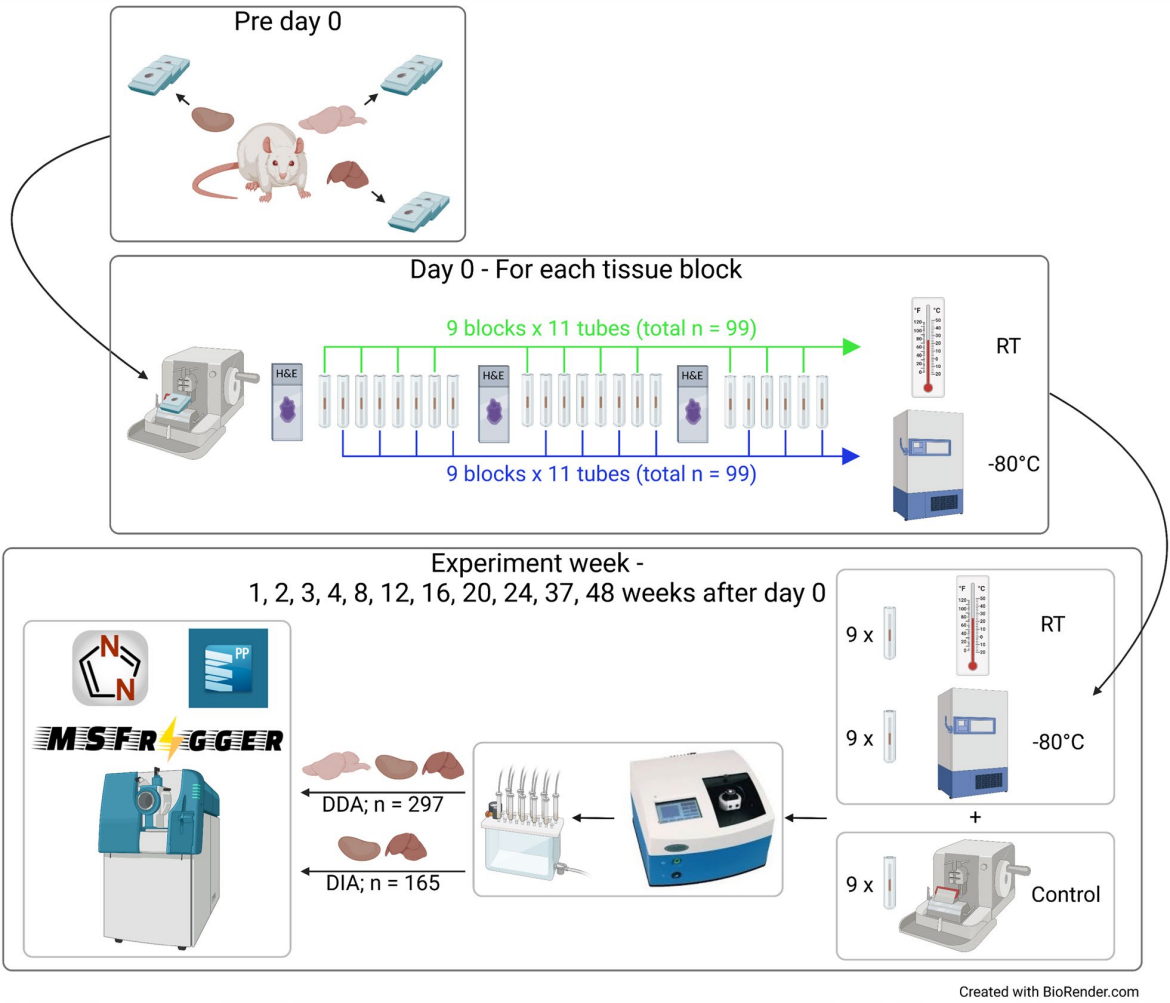
Summary of experimental design and workflow. A total of 9 FFPE blocks were prepared from rat brain, kidney and liver (each in triplicate). On day 0, each tissue block was sectioned to collect 3 × 4 µm sections (total n = 27) for H&E staining and 22 × 10 µm sections (total n = 198) for LC–MS analysis; an H&E slide was prepared after every eight 10 µm sections to track changes in tissue area. Half of the 10 µm sections (n = 99) were stored at RT and half (n = 99) were stored at – 80 °C. The samples were processed and analysed by LC–MS at 11 timepoints (1, 2, 3, 4, 8, 12, 16, 20, 24, 37 and 48 weeks after day 0). On each experiment day, one 10 µm section was freshly cut from each tissue block and used as control; the control sample was processed with day 0 sections that had been stored at RT and – 80 °C. All tissue digests (n = 297) were analysed on TripleTOF 6600 mass spectrometers in DDA mode after each experiment day. Kidney (n = 66) and liver (n = 99) digests were also analysed in DIA mode for quantitative analysis after 48 weeks

Tissue sections were prepared in triplicate from FFPE blocks of rat brain, kidney and liver and stored at RT or -80°C. They were processed to peptide digests along with the controls on 11 experiment days over 48 weeks. On each experiment day, 27 FFPE tissue sections were processed. A total of 297 samples were processed over the 48 weeks (Fig. 1). There was some variation in the peptide yield (µg of peptides per mm^2^ tissue) of freshly cut (control) and stored sections prepared over the 48-week period (Supp. Figure S1A). However, there was no specific trend indicating that this was related to the storage time or temperature. The observed differences in peptide yield are likely to be due to tissue content changes with block sectioning.

All samples (rat brain, kidney and liver) were analysed by LC–MS using DDA mode and the data was searched using ProteinPilot against a rat SWISSPROT canonical database. The digestion efficiency, as determined from the ProteinPilot search results, was above 80% for all tissue types regardless of their storage time or temperature (the data for only kidney is shown in Supp. Figure S1B). The number of proteins and peptides identified from DDA data at global 1% FDR showed no significant difference for time or temperature except for the stored samples from week 12 (Supp. Figure S1C and D). However, the number of peptide identifications was more affected by the performance of the instruments at the time of analysis than by section storage time or temperature. Common PTMs such as deamidation and oxidation were evaluated and despite some variability, no significant changes were observed in these PTMs (Supp. Figure S2).

**Fig. 2:**
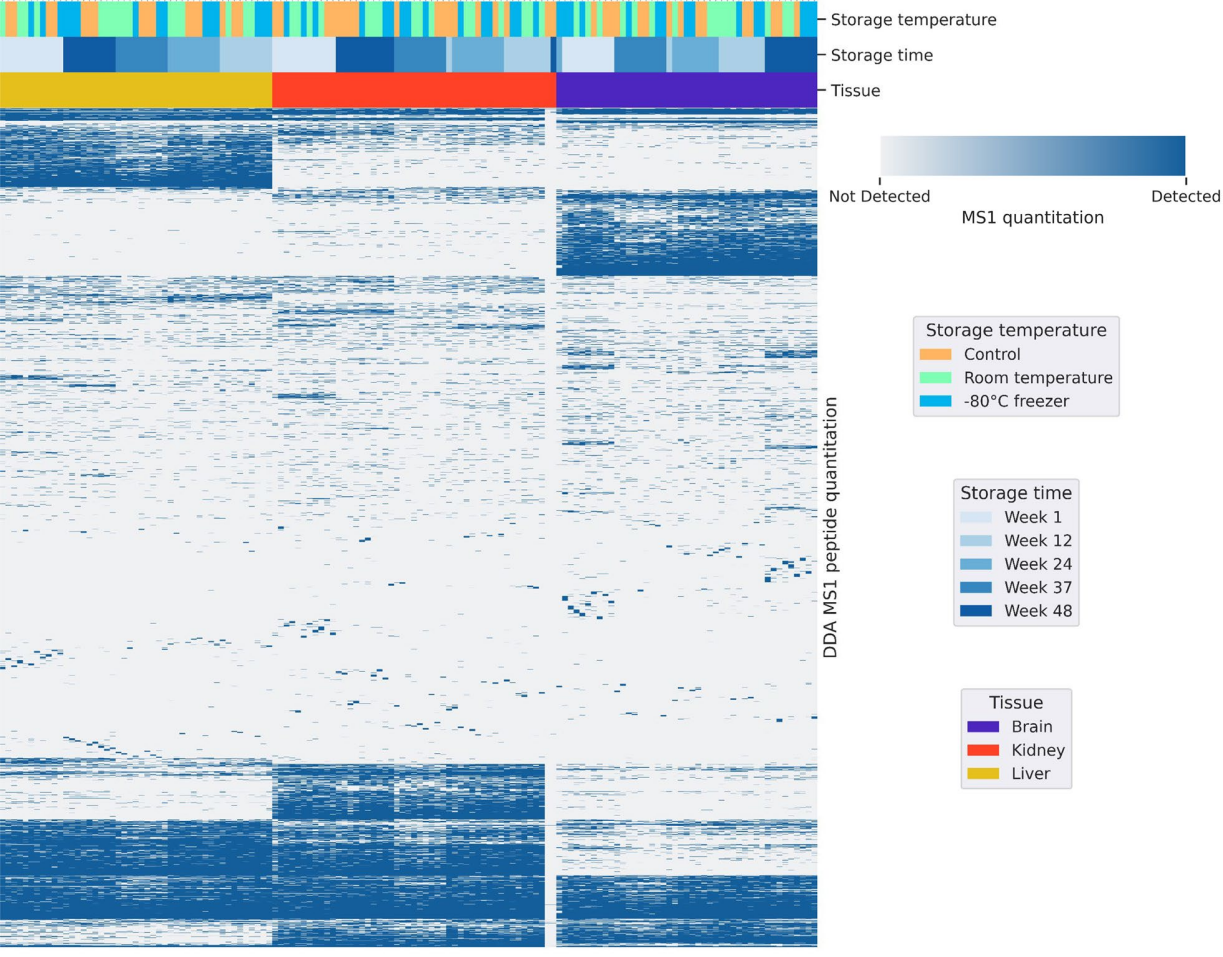
Label free quant (LFQ) analysis of DDA data from week 1, 12, 24, 37 and 48 (number of MS runs = 135) of rat brain, kidney and liver FFPE sections. Unsupervised hierarchical clustering heatmap of MS1 precursor quantitation showed grouping of samples according to their tissue type and storage time but not their storage temperature

Label-free quantitation (LFQ) of the peptides was performed, and the unsupervised hierarchical clustering analysis of the precursor (MS1) quantitation profiles showed three distinct clusters aligned to the three tissue types (Fig. 2). Within each cluster, samples analysed on the same experiment day aligned well, indicating their similarity, regardless of the storage temperature. The alignment of samples with the experiment day is likely caused by batch effects introduced by the sample preparation and instrument configuration, since the samples were processed and analysed as groups on different mass spectrometers at different timepoints. These results demonstrate that the proteomic profiles of the three tissues remained distinct across the experimental period, irrespective of storage time or storage temperature.

**Fig. 3:**
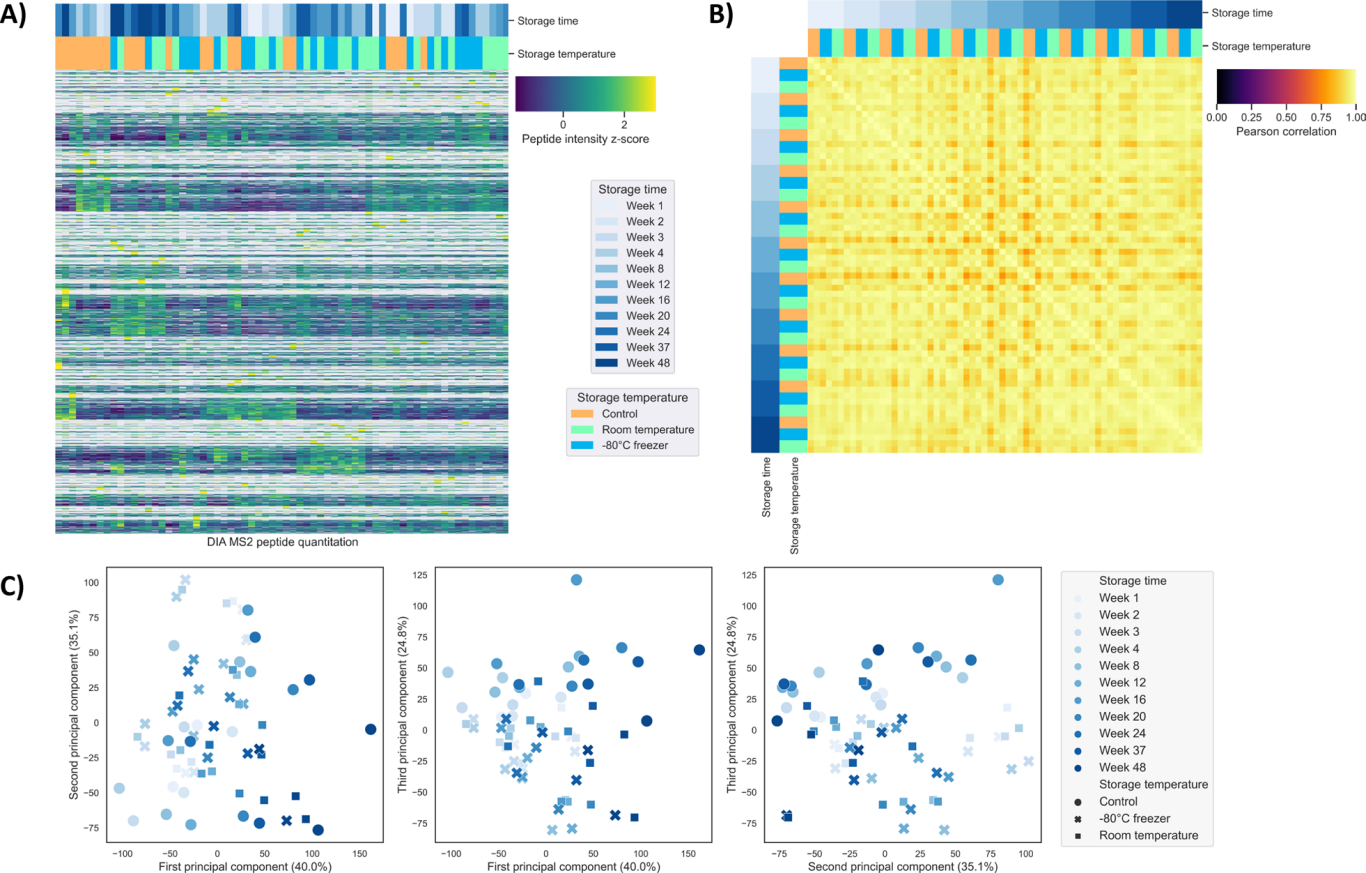
DIA analysis of rat kidney FFPE sections (number of MS runs = 66): **A** unsupervised hierarchical clustering heatmap of the peptide intensity z-score (total number of peptides = 34,781) showed no grouping of samples according to their storage time or temperature; **B** Pearson correlation showed good correlation among the samples; and **C** principal component analysis showed some clustering at principal component 3 most likely due to the position of sections taken from within the tissue blocks

Selected peptide digests from the full cohort were analysed using DIA with DIA-NN (n = 165) for a series of quantitative analyses, while other digests were depleted during DDA analysis. The spectral library was generated from the MSFragger closed search, with the nine modifications common to FFPE samples being included in the library. A z-score was calculated and used to compare the intensity of each peptide to the mean intensity across samples in the kidney (n = 66) and liver (n = 99) subsets. Unsupervised hierarchical clustering analysis of the peptides did not show grouping of samples by their storage time or storage temperature (Fig. 3A, Supp. Figure S3A). Good correlation was observed among the samples, suggesting that the storage time or storage temperature did not detectably affect the proteomic features of FFPE sections (Fig. 3B, Supp. Figure S3B). However, the controls show some separate clustering from the samples that were stored at RT and -80 °C at the third principal component of the principal component analysis (PCA) of kidney samples, but this was not observed in liver samples (Fig. 3C, Supp. Figure S3C). The differentiation could be due to difference in tissue content and where the sections were taken from within the tissue blocks. All the sections stored at RT and − 80 °C were cut at the beginning of the experiment while the control sections were freshly cut on the day of the experiment, hence located further into the blocks. Therefore, the stored samples would be expected to exhibit more similarities among themselves compared to the control samples. This is supported by the observation that the control samples from earlier weeks had closer proximity in the PCA to the stored samples than that of later weeks. To further determine if FFPE tissue sections are stable over the 48 weeks, paired *t*-test was performed and the *p*-values were adjusted using Bonferroni method. Three out of 34,781 kidney peptides (0.008%) and 162 out of 37,104 liver peptides (0.44%) had *p*-values less than 0.05, both well below the 1% FDR. This indicates that storage time did not have significant impacts on the LC–MS analysis of FFPE samples.

**Fig. 4:**
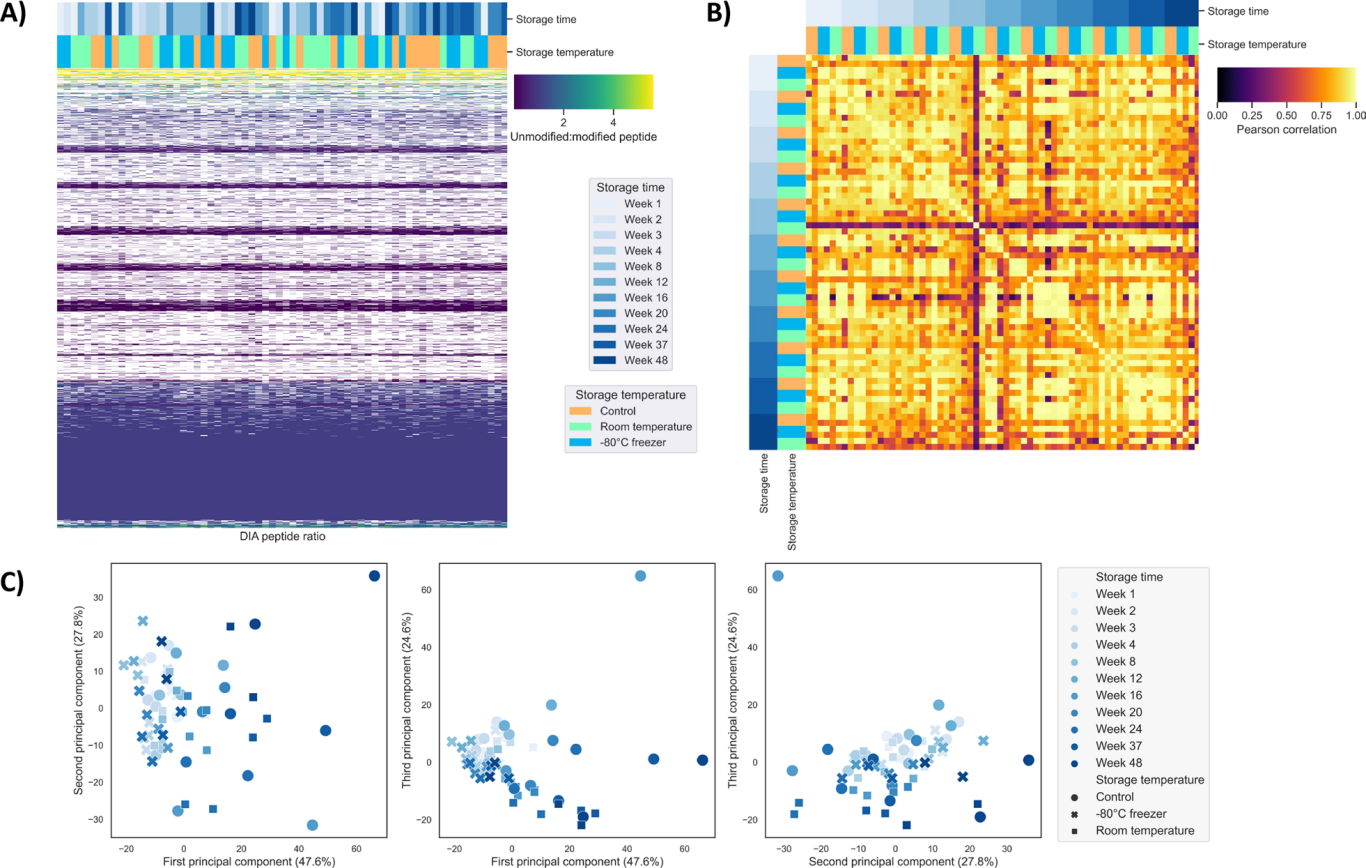
DIA analysis of rat kidney FFPE sections (number of MS runs = 66): **A** unsupervised hierarchical clustering heatmap of the ratio of modified peptides to their unmodified counterparts (number of peptides = 6223) showed no grouping of samples according to their storage time or temperature; **B** Pearson correlation showed good correlation among the samples; and **C** principal component analysis showed partial clustering

As modifications can potentially accumulate with FFPE tissue block or section storage, peptides with modifications that also had data for their corresponding unmodified counterparts were quantitatively analysed using DIA data for possible changes over time or with the storage temperature. The ratio of the modified peptides to their unmodified counterparts was determined from rat kidney and liver DIA data. Changes in peptide modifications in these pairs were not detectably affected by the storage time or temperature. The unsupervised hierarchical clustering analysis did not group the samples by their storage time or temperature (Fig. 4A, Supp. Figure S4A), and the samples had good correlation (Fig. 4B, Supp. Figure S4B). Partial clustering was observed in PC1 versus PC3 in kidney samples as the samples stored at − 80°C were separated from the other samples (Fig. 4C). Similarly, partial clustering was also observed in the PCA of liver samples where the samples stored at − 80°C were grouped (Supp. Figure S4C). These variations were likely introduced by the content of tissue within the sections and type of proteins present in the samples as different proteins react differently with formaldehyde over time [1, 36]. It is also possible that storing cut sections at − 80°C may have some impacts on the degree of modifications that occur during storage, but this requires further investigation.

**Fig. 5:**
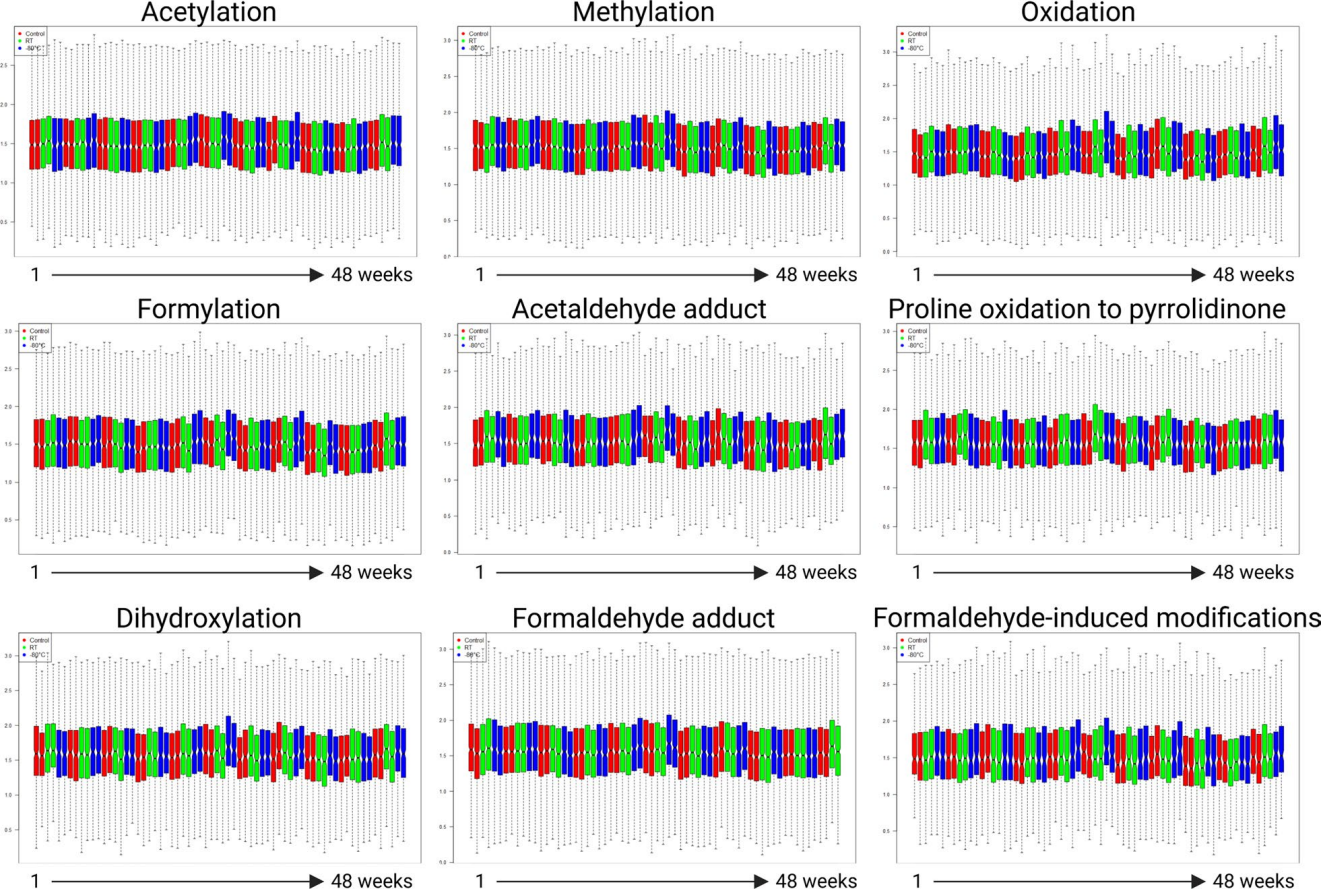
FFPE-specific post-translational modifications in rat kidney FFPE sections. Nine modifications that have been reported to be associated with FFPE samples were monitored for quantitative changes using DIA data. Samples were grouped by the storage temperature (control, RT or – 80 °C) and from week 1 to week 48 (left to right)

Formaldehyde-induced cross-links and modifications are a known concern with molecular analyses of FFPE samples. Formaldehyde reacts mainly with basic amino acid residues such as arginine and lysine, therefore basic proteins are likely to have more cross-links than acidic proteins [13, 24, 37–39]. Increased oxidation, particularly of methionine, occurs in long-term archived tissues [24]. Therefore, nine modifications that are commonly reported in FFPE samples from other studies [24, 40, 41], as well as the MSFragger open search of DDA data (Table 1), were quantitatively determined for their changes over the storage time or temperature. Changes were monitored for: acetylation of *N*-termini; methylation of *N*-termini and lysine; oxidation of methionine and proline; formylation of *N*-termini and lysine; acetaldehyde adducts on *N*-termini; proline oxidation to pyrrolidinone; dihydroxylation of methionine and proline; formaldehyde adducts on *N*-termini, arginine and lysine; and formaldehyde-induced modifications of the *N*-termini and lysine. The abundance of these common FFPE modifications was relatively consistent in both kidney and liver samples, regardless of their storage time or storage temperature (Fig. 5, Supp. Figure S5).

To further investigate long-term storage impacts, FFPE tissue block age was examined. Tissue sections were prepared from 77 oropharyngeal tumour FFPE blocks that had been stored between 4 and 13 years and processed to peptide digests for LC–MS analysis using DIA. The samples cut from older blocks did not have lower peptide yield, or a lower number of quantified proteins or peptides (Supp. Figure S6). There was no trend that may have related these measurements to the age of the tissue blocks. Quantitative analysis of DIA data for common FFPE modifications in these samples was performed using DIA-NN against a cohort-specific spectral library. No modifications markedly changed across the samples in association with age of the blocks (Supp. Figure S7). Unsupervised hierarchical clustering analysis and PCA did not group the samples by their block age (Supp. Figure S8). This suggests that long-term storage of archived FFPE blocks, prior to their sectioning, does not have adverse impacts on the detectable proteome for at least 10 years.

Our results are consistent with other studies that have shown that archival FFPE tissue blocks stored up to 10 years are suitable for proteomic analysis with a comparable number of proteins and peptides identified or quantified [1, 3, 20, 42]. The quantity and quality of the proteins and peptides extracted from FFPE samples could be affected by pre-analytical factors including tissue size, tissue handling, inconsistencies in preservation protocols between different laboratories and the storage conditions (temperature and humidity). Long-term storage of FFPE samples can cause signal reduction in IHC studies due to loss of immunoreactivity (i.e., protein denaturation) but not protein degradation. Therefore, LC–MS analysis should not be affected by the storage time of FFPE samples [21]. Another study also concluded that FFPE is the preferable preservation method relative to FF for its cost effectiveness and long-term storage of cryo-preserved samples can affect the quality of proteins extracted from the samples [2, 43].

It cannot be ruled out that identification of certain proteins could be affected by the storage time, although the overall proteome did not show obvious changes [20, 22]. For example, low abundance proteins could become more difficult to retrieve and quantify from FFPE tissue blocks that are more than 10 years old [22]. Similarly, full-length proteins are more difficult to extract from older FFPE blocks due to the continuation of formaldehyde-induced cross-linking over time [1]. It is hypothesised that proteins react with formaldehyde to different extents. Larger proteins with greater lysine content are more likely to be extensively cross-linked by formaldehyde and are harder to detect using MS compared to small proteins with low lysine content [36]. Most proteins extracted from FFPE tissues have low to medium molecular weight (40–60 kDa) [1]. Despite that, protein biomarker candidates have been successfully identified from FFPE samples with the same proteins identified in matching FF samples [19]. Another study also reported two medium to low abundance proteins being quantified using multiple reaction monitoring (MRM) assays from a 15 year old archived sample [43]. Therefore, it is promising that FFPE tissue blocks stored in biobanks are suitable for proteomic analysis, biomarker discovery and validation. It is beneficial to use FFPE samples for large-scale retrospective studies given the large repositories of FFPE samples and the presence of their associated pathology reports and clinical data.

## Conclusions

The use of FFPE samples for proteomic analysis was previously thought to be limited by formaldehyde-induced cross-links and modifications. Current sample preparation technologies have overcome these problems and FFPE sample preparation improved such that they may now be considered a good resource readily available in biobanks worldwide, are more cost effective to store and transport, and are safe to handle.

In this study, the effect of storage time and storage temperature on FFPE cut sections for proteomic analysis was investigated. Tissues were harvested from a single animal, which may limit the biological variability observed in the results. The sections were cut and stored at RT or − 80 °C for up to 48 weeks before they were processed and analysed by LC–MS. The tissue sections were stored inside enclosed tubes, therefore they had minimal exposure to the outside air. The surface of the tissue blocks from which sections were taken was more likely to be exposed to air, but they were trimmed before the control sections were taken. The temperature and humidity of the room were controlled and the fluctuation was minimal.

Stored cut sections gave similar results to control sections that were freshly cut on the experiment day. No significant differences were detected in either the overall proteome or FFPE-related modifications in FFPE samples that were sectioned and stored for later proteomic analysis at different temperature or storage time of almost a year. Similar observations were made with both DDA and DIA data. In contrast to their use for immunohistochemistry, stored FFPE tissue sections are stable and suitable for proteomic studies for at least 48 weeks from the time of their sectioning, while their parent blocks are similarly stable for at least 10 years.

## Supplementary Information

Supplementary Material 1. Figure S1. No impact of storage time or temperature on overall peptide and protein recovery. A) Peptide yield (µg of peptides per mm^2^ of tissue); B) digestion efficiency (the percentage of peptides ending in arginine [R] or lysine [K]); C) numbers of proteins identified; and D) number of peptides identified. The data is from DDA analysis of rat kidney FFPE sections. Samples of each storage type of each week were represented in the average of the triplicate ± SD and *p* < 0.001 (**). Figure S2. No impact of FFPE section storage time or temperature on common post-translational modifications. The data is shown for nine common modifications that can occur in biological samples identified by DDA analysis of rat kidney FFPE sections. The letter in the bracket indicates the amino acid residue that is affected by the modification. The y-axis represents the percentage of peptides that were affected by the modification. Samples of each storage type of each week were represented in the average of the triplicate ± SD and *p* < 0.05 (*). Figure S3. DIA analysis of rat liver FFPE sections (number of MS runs = 99): A) unsupervised hierarchical clustering heatmap of the peptide intensity z-score (total number of peptides = 37,104) showed no grouping of samples according to their storage time or temperature; B) Pearson correlation showed good correlation among the samples; and C) principal component analysis showed no clustering of samples. Figure S4. DIA analysis of rat liver FFPE sections (number of MS runs = 99): A) unsupervised hierarchical clustering heatmap of the ratio of modified peptides to their unmodified counterparts (number of peptides = 7,212) showed no grouping of samples according to their storage time or temperature; B) Pearson correlation showed good correlation among the samples; and C) principal component analysis showed partial clustering. Figure S5. FFPE-specific post-translational modifications in rat liver FFPE sections. Nine PTMs that have been reported to be associated with FFPE samples were monitored for quantitative changes using DIA data. Samples were grouped by the storage temperature (control, RT or -80°C) and from week 1 to week 48 (left to right). Figure S6. No impact of FFPE tissue block age on overall peptide and protein recovery. A) Peptide yield; B) proteins quantified; and C) peptides quantified. The data is from DIA analysis of 77 FFPE sections prepared from oropharyngeal tumour blocks aged from 4 to 13 years. Figure S7. FFPE-specific post-translational modifications in FFPE sections (n = 77) prepared from oropharyngeal tumour blocks aged from 4 to 13 years. Nine modifications that have been reported to be associated with FFPE samples were monitored using DIA analysis. Samples were grouped by the age of the FFPE blocks, from 4 to 13 years (left to right). Figure S8. DIA analysis of FFPE sections (n = 77) prepared from oropharyngeal tumour blocks aged from 4 to 13 years: A) unsupervised hierarchical clustering heatmap of the peptide intensity z-score (number of peptides = 81,016) and B) principal component analysis showed no grouping of samples according to their block age.

## Data Availability

The mass spectrometry proteomics data have been deposited to the ProteomeXchange Consortium via the PRIDE [35] partner repository with the dataset identifier PXD054596.

## Abbreviations

DDA: Data-dependent acquisition
DIA: Data-independent acquisition
DIA-NN: Data-Independent Acquisition by Neural Networks
FA: Formic acid
FDR: False discovery rate
FF: Fresh frozen
FFPE: Formalin-fixed paraffin-embedded
H&E: Haematoxylin and eosin
IHC: Immunohistochemistry
IOA: Iodoacetamide
LC–MS: Liquid chromatography-mass spectrometry
LFQ: Label-free quantitation
MRM: Multiple reaction monitoring
PCA: Principal component analysis
PTM: Post-translational modification
RT: Room temperature
SDC: Sodium deoxycholate
TCEP: Tris(2-carboxyethyl)phosphine
TEAB: Triethylammonium bicarbonate
