# Supplementary material for: The effect of storage time and temperature on the proteomic analysis of FFPE tissue sections
Source: Clin Proteomics. 2025 Feb 5;22:5. doi: 10.1186/s12014-025-09529-5 (PMC11796064; doi:10.1186/s12014-025-09529-5)
Supplement: Supplementary file 1 — Supplementary Material 1. Figure S1. No impact of storage time or temperature on overall peptide and protein recovery. A) Peptide yield (µg of peptides per mm2 of tissue); B) digestion efficiency (the percentage of peptides ending in arginine [R] or lysine [K]); C) numbers of proteins identified; and D) number of peptides identified. The data is from DDA analysis of rat kidney FFPE sections. Samples of each storage type of each week were represented in the average of the triplicate ± SD and p < 0.001 (**). Figure S2. No impact of FFPE section storage time or temperature on common post-translational modifications. The data is shown for nine common modifications that can occur in biological samples identified by DDA analysis of rat kidney FFPE sections. The letter in the bracket indicates the amino acid residue that is affected by the modification. The y-axis represents the percentage of peptides that were affected by the modification. Samples of each storage type of each week were represented in the average of the triplicate ± SD and p < 0.05 (*). Figure S3. DIA analysis of rat liver FFPE sections (number of MS runs = 99): A) unsupervised hierarchical clustering heatmap of the peptide intensity z-score (total number of peptides = 37,104) showed no grouping of samples according to their storage time or temperature; B) Pearson correlation showed good correlation among the samples; and C) principal component analysis showed no clustering of samples. Figure S4. DIA analysis of rat liver FFPE sections (number of MS runs = 99): A) unsupervised hierarchical clustering heatmap of the ratio of modified peptides to their unmodified counterparts (number of peptides = 7,212) showed no grouping of samples according to their storage time or temperature; B) Pearson correlation showed good correlation among the samples; and C) principal component analysis showed partial clustering. Figure S5. FFPE-specific post-translational modifications in rat liver FFPE sections. Nine PTMs that h [file 12014_2025_9529_MOESM1_ESM.pptx]

## Slide 1
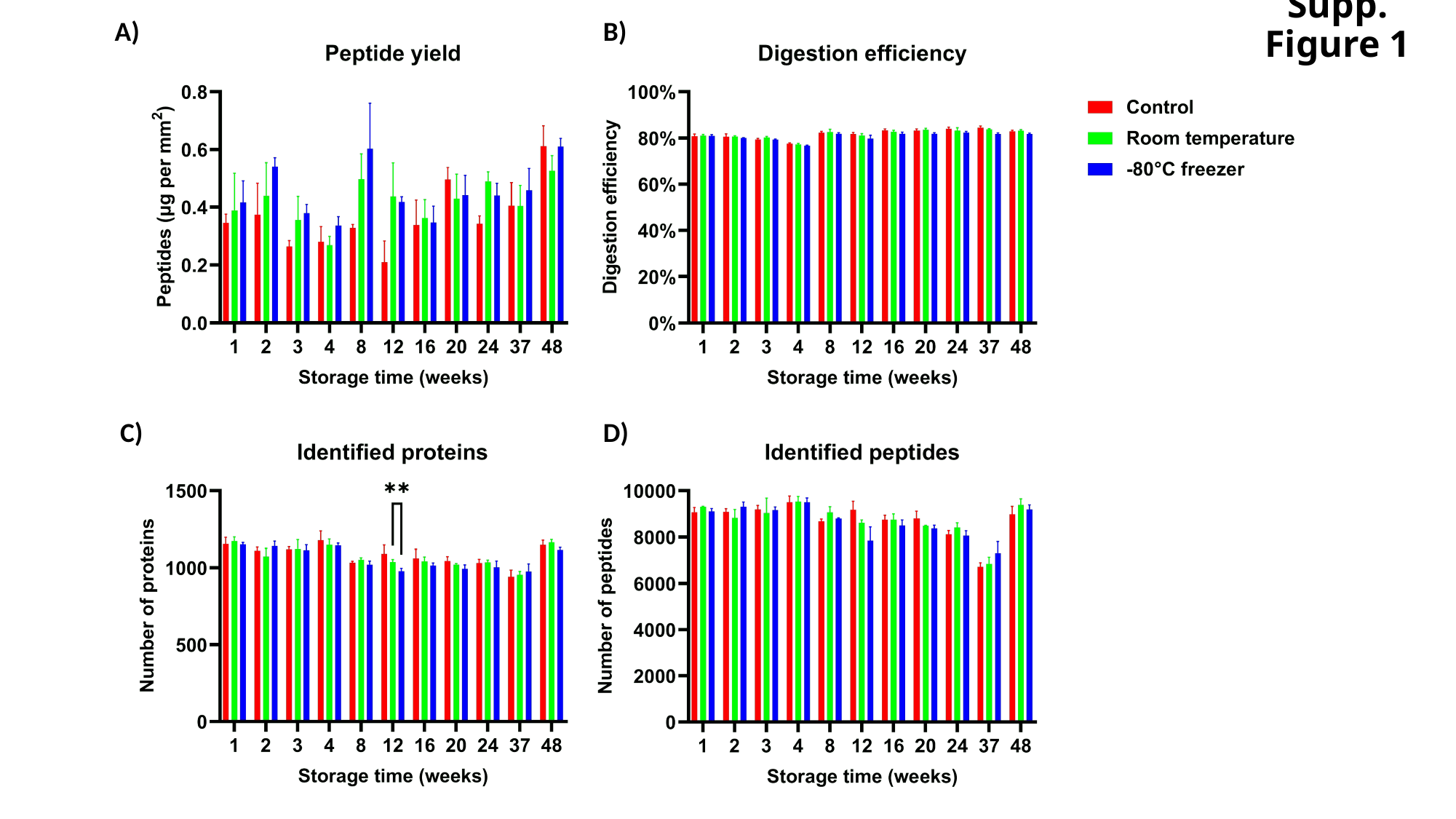

Supp. Figure 1
A)
B)
C)
D)

## Slide 2
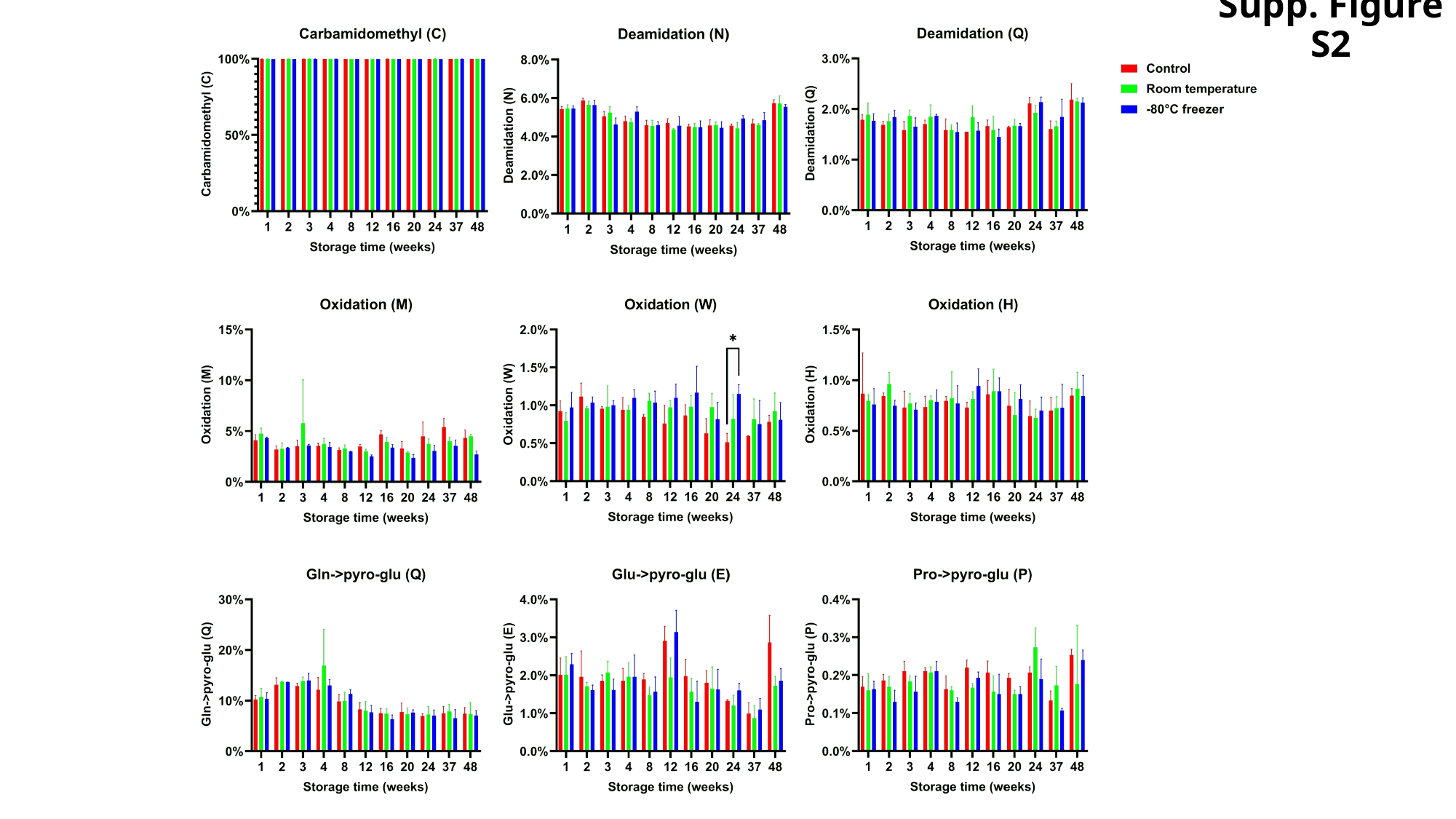

Supp. Figure S2

## Slide 3
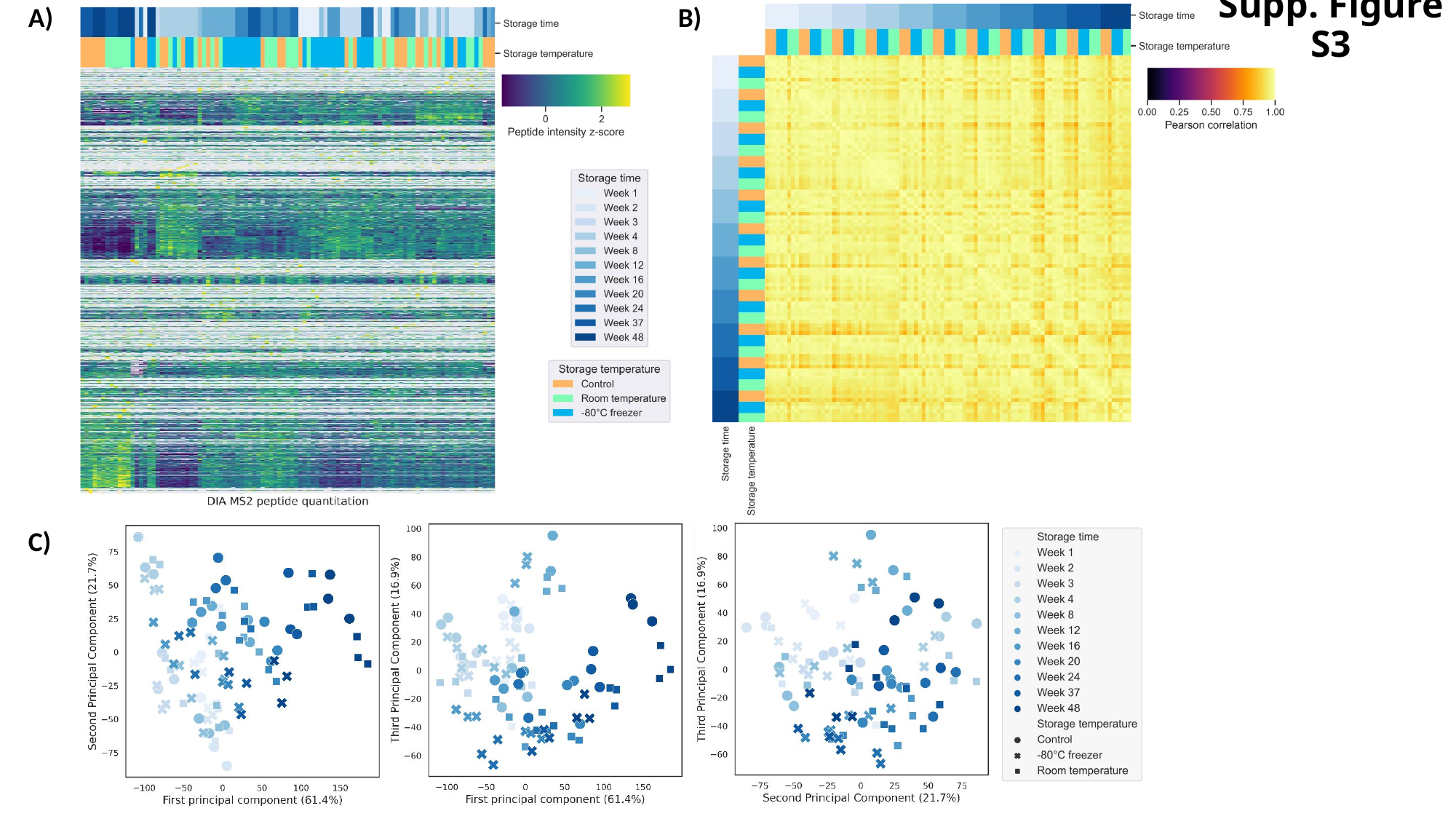

A)
B)
C)
Supp. Figure S3

## Slide 4
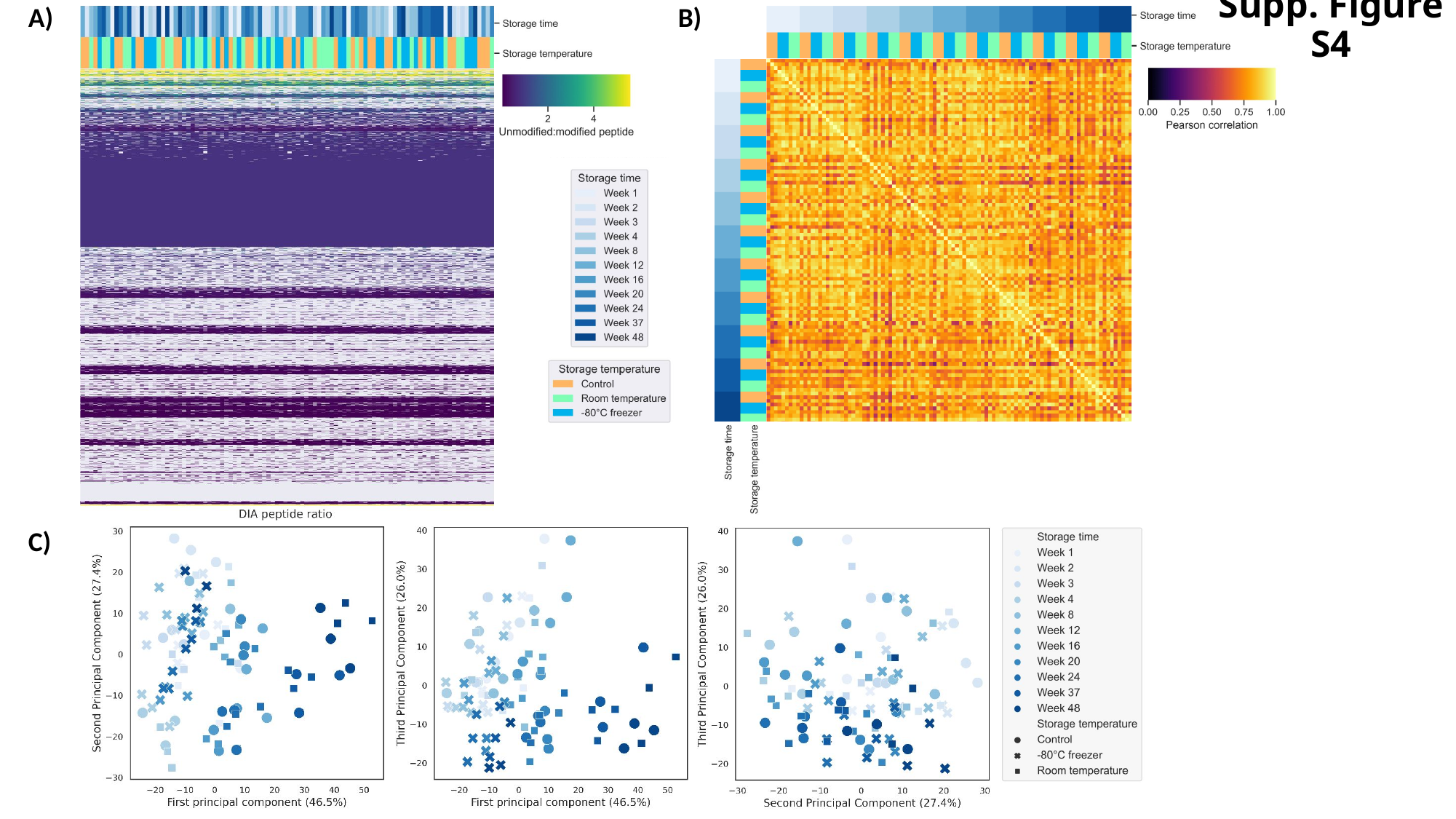

A)
B)
Supp. Figure S4
C)

## Slide 5
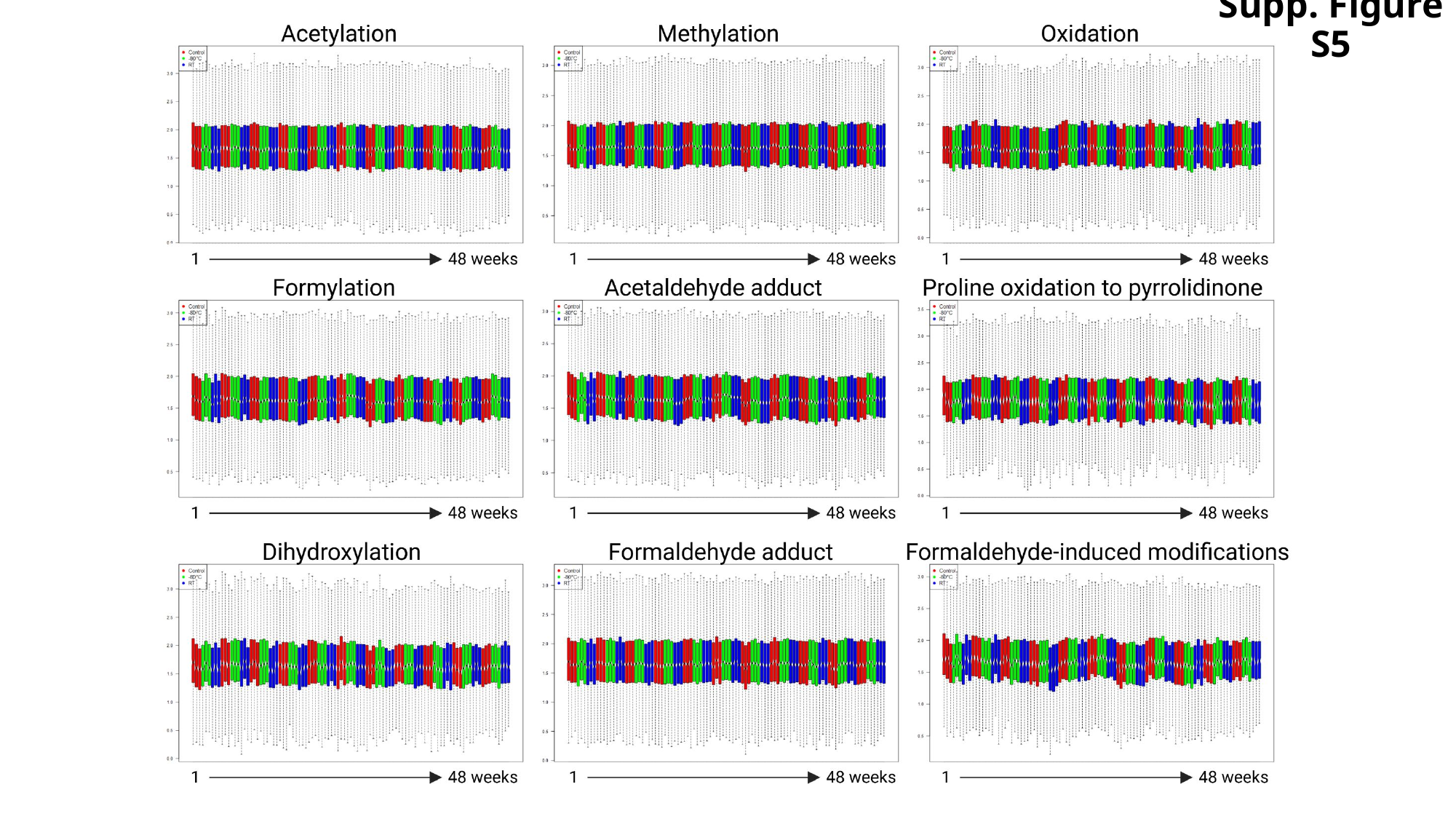

Supp. Figure S5

## Slide 6
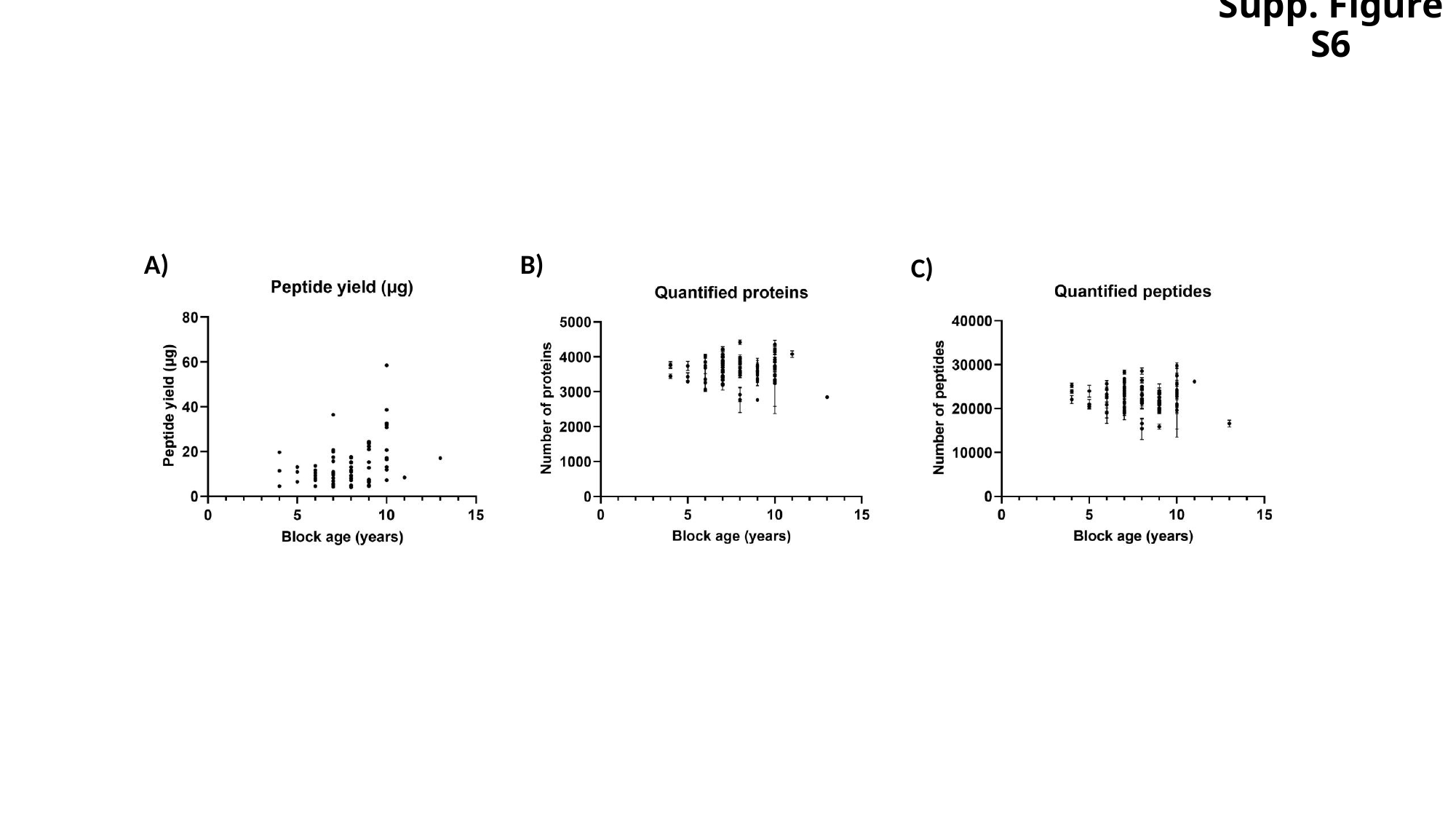

Supp. Figure S6
A)
B)
C)

## Slide 7
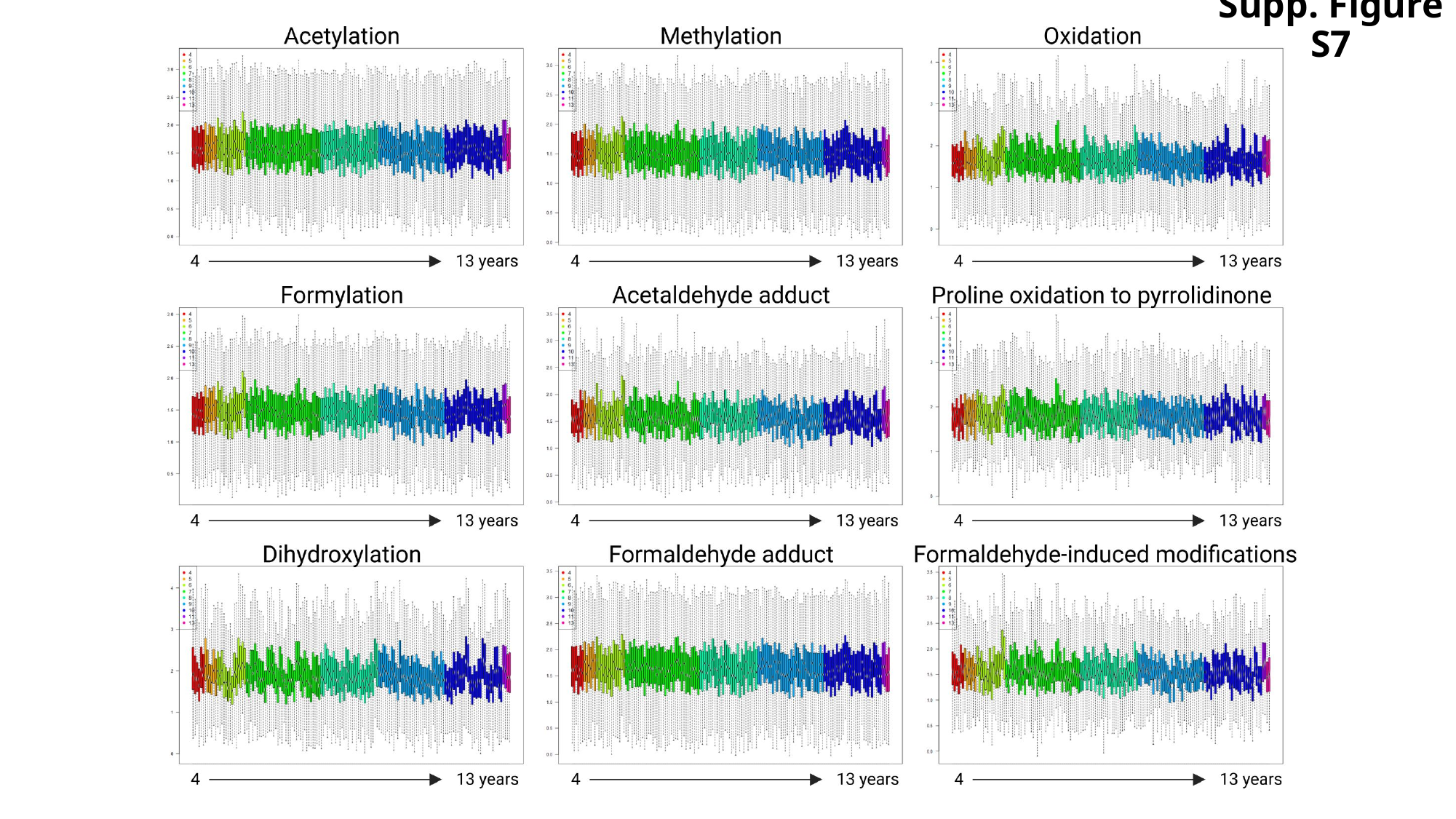

Supp. Figure S7

## Slide 8
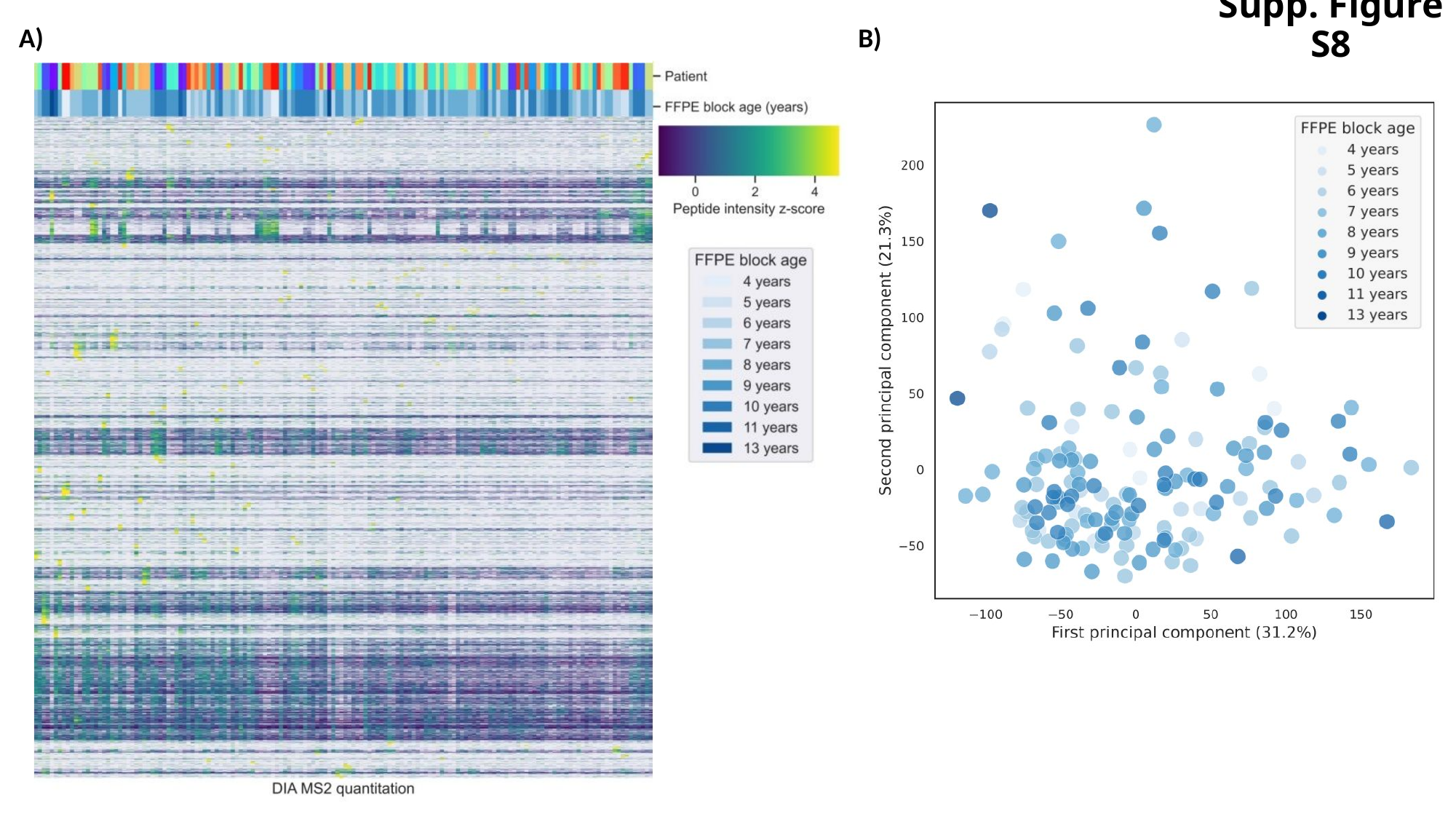

Supp. Figure S8
A)
B)
